# Nature-based virtual reality intervention to manage stress in family caregivers of allogeneic hematopoietic stem cell transplant recipients: a two-phase pilot study protocol

**DOI:** 10.3389/fpsyt.2024.1295097

**Published:** 2024-03-07

**Authors:** Lena J. Lee, Elisa H. Son, Nicole Farmer, Chantal Gerrard, Ralph Thadeus Tuason, Li Yang, Julie Kohn-Godbout, Cory Stephens, Eun-Shim Nahm, Leslie Smith, Steve Risch, Gwenyth R. Wallen

**Affiliations:** ^1^ National Institutes of Health, Clinical Center, Translational Biobehavioral and Health Disparities (TBHD), Bethesda, MD, United States; ^2^ University of Maryland Baltimore, School of Nursing, Baltimore, MD, United States; ^3^ National Institutes of Health, Clinical Center, Nursing Department, Bethesda, MD, United States

**Keywords:** virtual reality, caregivers, hematopoietic stem cell, stress, biomarkers

## Abstract

Millions of family members and/or friends in the U.S. serve as unpaid caregivers for individuals with chronic conditions, such as cancer. Caregiving for someone undergoing an intense allogeneic hematopoietic stem cell transplant (HSCT) is particularly demanding, with accompanying physical and psychological stress. Increased stress and stress-related symptoms could make it difficult for caregivers to fulfill their roles and could negatively impact the health status and quality of life of themselves and the recipients. Virtual reality (VR) is a promising technology increasingly used for treatment and wellness in various medical settings. There is growing evidence that studies have reported the positive effects of the VR intervention in managing and reducing stress among diverse populations in various clinical scenarios; however, no published studies have focused on family caregivers of patients with cancer. The study aims to assess the feasibility and acceptability of a four-week nature-based VR intervention and to examine the effectiveness of the VR intervention on stress in HSCT caregivers. This study comprises two phases. Phase I of the study will be a single-arm pre-post design focused on assessing the feasibility and acceptability of the VR intervention. Phase II of the study will be a prospective randomized controlled group design to examine the effectiveness of the VR intervention on perceived stress. Adults (≥ 18 years) who serve as primary caregivers for a person who will undergo an allogeneic HSCT will be recruited. Fifteen participants will be enrolled for Phase I and 94 participants for Phase II (Active VR arm N=47; Sham VR arm N=47). The nature-based immersive VR program contains 360° high-definition videos of nature scenes along with nature sounds through a head-mounted display (HMD) for 20 minutes every day for four weeks. Primary outcome is perceived stress measured by the Perceived Stress Scale. Secondary/exploratory outcomes are stress-related symptoms (e.g., fatigue, sleep disturbance) and physiological biomarkers (e.g., cortisol, alpha-amylase). The importance and innovativeness of this study consist of using a first-of-its-kind, immersive VR technology to target stress and investigating the health outcomes assessed by validated objective biomarkers as well as self-report measures of the nature-based intervention in the caregiver population.

**Clinical trial registration:**

ClinicalTrials.gov, identifier NCT 05909202.

## 1 Introduction

Hematopoietic stem cell transplant (HSCT), also called bone marrow transplant, is a procedure to treat malignancies and non-malignant conditions by replacing dysfunctional bone marrow with healthy stem cells from the patient’s own body (autologous) or a donor (allogeneic) (1). Since 2016, an average of more than 22,000 HSCTs have been performed in the United States each year (2). Caring for a HSCT recipient is burdensome as the recipient is at risk for complications (e.g., graft versus host disease and cytopenia) and requires extensive support during and after treatment (3). Family caregivers of HSCT recipients may feel rewarded while taking on caregiving tasks, but they may also experience stress and stress-related symptoms (4, 5). Frequently reported symptoms include fatigue, sleep disturbance, depression, anxiety, and impaired cognition, which tend to occur concurrently (4–7). Stress and symptoms experienced by HSCT caregivers can negatively impact health outcomes for both caregivers and the recipient. However, there are limited resources available to address stress and symptoms in HSCT caregivers.

Virtual reality (VR) is a promising technology that can be used to develop interventions to manage stress and symptoms in HSCT caregivers. VR is a type of extended technology, defined as “a simulated three-dimensional environment that enables users to explore and interact with a virtual surrounding perceived through the user’s senses (8).” People mainly interact with virtual environments using special equipment such as a headset with a screen inside and a controller with sensors fitted (9). As VR has become more immersive, affordable and portable, various areas, including education, entertainment, and healthcare, are using this technology (10, 11). The application of VR in healthcare includes training for students and medical professionals and treatment and wellness uses such as stress and symptom improvement (e.g., pain management) (12). Many studies that developed VR-based interventions simulated nature environments (e.g., forest, beach, lake) on a virtual platform and tested the effects of experiencing this nature on health-related outcomes (10, 13, 14). These studies have shown positive effects in reducing pain, stress, and unpleasant psychological symptoms (10, 13, 14).

Nature experience in VR might also help relieve stress and symptoms in HSCT caregivers. However, there are limited studies examining the effectiveness of VR-based interventions in family caregiver populations. Existing intervention studies to address stress and symptoms in family caregivers often took a mindfulness approach and included yoga, breathing exercise, and meditation as components of the program (15–17). These intervention programs necessitated a lot of time and effort from caregivers who may find it difficult to be away from their care recipients. Stress reduction interventions using VR technology may allow family caregivers to use the intervention at their desired location and time. In addition, integrating biomarkers into VR intervention studies for family caregivers would allow for a more comprehensive assessment of caregivers’ response to VR interventions than relying solely on self-report measures (18, 19). As caregiving is a potential stressor that may contribute the development of cardiometabolic and inflammatory disease (20, 21), it is important to include biomarkers that can objectively measure stress responses (e.g., cortisol, α-amylase, osteocalcin, Oxytocin), and cardiometabolic (e.g., lipoprotein particle profile) and immune function (e.g., cytokine) in cancer caregiving research.

**Figure 1:**
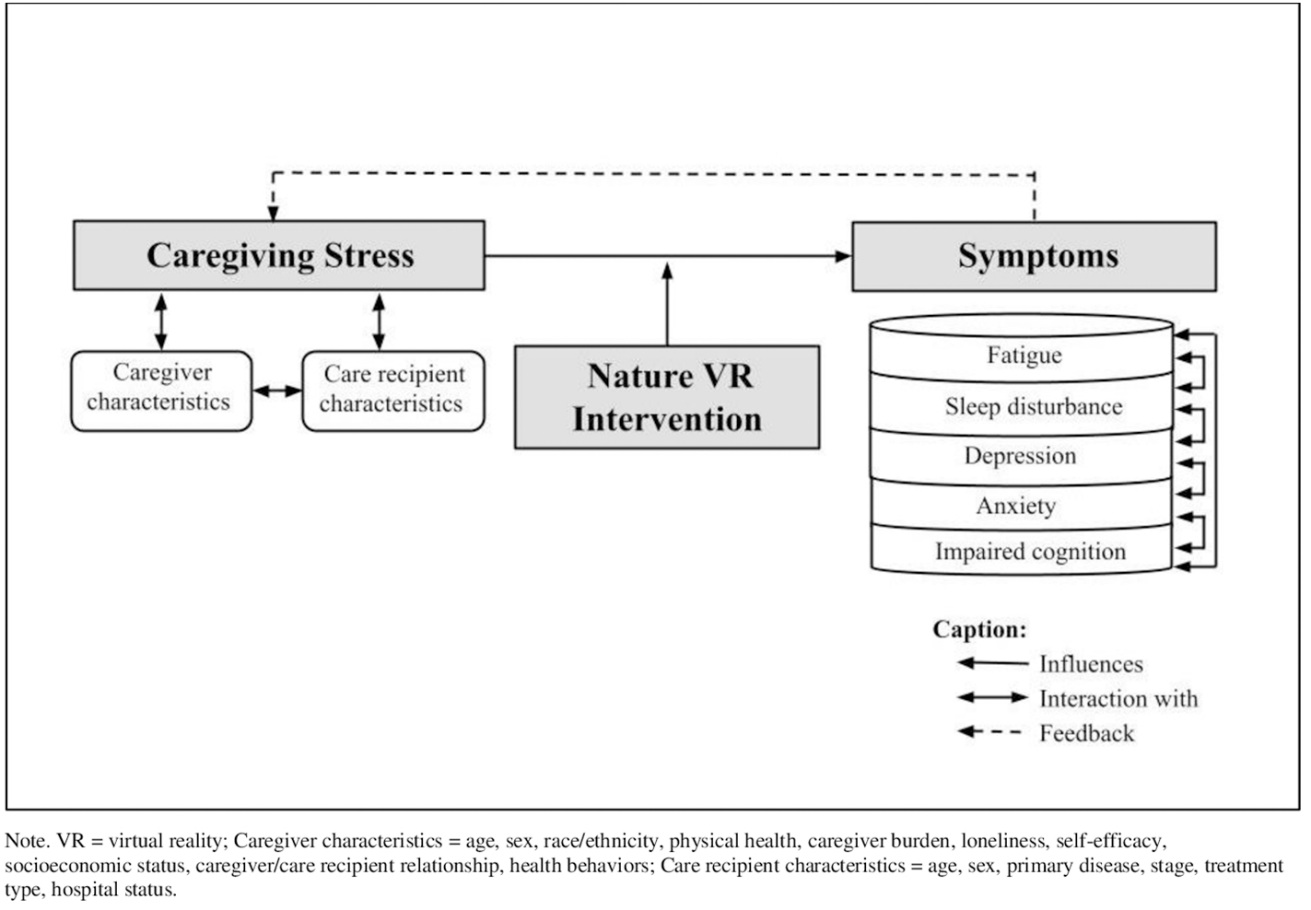
Conceptual model of factors influencing caregiving stress and symptoms. VR, virtual reality.

This research is guided by a conceptual model based on the Attention Restoration Theory (ART; 22, 23) and the Theory of Unpleasant Symptoms (TOUS; 24). According to the ART, experiencing restorative environments induces involuntary attention engagement, leading to the restoration of directed attention and the relief from directed attentional fatigue (22, 23). The ART explains that interaction with nature environments allows directed attention to get rest and recover while using involuntary attention, which can be beneficial for performing tasks and reducing stress and negative moods. Restoration of directed attention primarily occurs in nature environments but may also occur in some urban settings with sufficient restorative qualities, such as historical sites or museums (22, 23, 25). The TOUS assumes that symptoms can occur individually or concurrently, and these symptoms form a feedback loop that also affects individual factors that contribute to the symptom experience (24, 26). In our conceptual model (see **Figure 1**), caregiving stress leads to caregivers’ multiple symptoms, including fatigue, sleep disturbance, depression, anxiety, and impaired cognition. Caregiving stress is closely related to caregiver characteristics and care recipient characteristics. Each symptom experienced by caregivers likely interacts with each other.

A growing body of literature reveals that a pleasant and immersive nature virtual environment may contribute to facilitating relaxation and stress management compared to urban scenes (27–29). These benefits from being exposed to virtual nature are generally consistent with those from actual nature (27). However, controversy still exists on the advantage of virtual immersion in nature versus traditional 2D mediums (non-immersive) (27). Furthermore, no published studies have examined the effectiveness of nature experience in VR, focusing on family caregivers of cancer patients. Building upon the feasibility study with the nature-based immersive VR intervention and the comparison condition (non-immersive VR program) have been developed in HSCT caregivers. We aim (1) to evaluate the feasibility and acceptability of the nature-based immersive VR program; (2) to examine the effectiveness of a nature-based immersive VR program on stress compared to a non-immersive nature-based VR in HSCT caregivers; and (3) to explore relationships among variables, including but not limited to symptoms, HSCT caregiver characteristics, HSCT recipient characteristics, and biomarkers. We propose that the nature-based VR intervention will effectively relieve caregiving stress in HSCT caregivers.

## 2 Methods and analysis

### 2.1 Design

The proposed study is a single site, two-phase study targeting allogeneic HSCT caregivers. The Phase I of the study is a single-arm pre-post design to assess the feasibility and acceptability of the nature-based immersive VR program in HSCT caregivers. The Phase II of the study is a prospective randomized controlled trial (RCT) design to examine the effectiveness of the nature-based immersive VR program on stress and symptoms compared to a nature-based non-immersive VR program in the target population. This study followed the Consolidated Standards of Reporting Trials (CONSORT) reporting guidelines for RCTs (**Supplementary Table S1**). In Phase I, all caregiver participants will be given the nature-based immersive VR program. We hypothesize that the majority of caregiver participants will perceive the nature-based immersive VR program as usable, safe, and restorative and show improved levels of stress and symptoms at Time 4 (Day 28) compared to Time 0 (Day 0). In Phase II, caregiver participants will be randomly assigned to one of two groups: nature-based immersive VR program (Active VR) and nature-based non-immersive VR program (Sham VR). We hypothesize that caregiver participants assigned to Active VR will show improved levels of stress and symptoms at Time 4 compared to those assigned to Sham VR. If we identify any weaknesses or gaps in the results of Phase I, such as dissatisfaction with the program (e.g., content, length of the video, difficulty in use) and discomfort with the headset, we plan to make appropriate adjustments prior to initiating Phase II.

### 2.2 Participants

The research protocol will include adults (18 years and older) who serve as a primary caregiver for an adult (18 years and older) planning to undergo an allogeneic HSCT at the NIH Clinical Center. If more than one caregiver is planned for the transplant recipient during the transplant phase, only one primary caregiver will be eligible to participate in this study. Phase I will enroll up to 15 caregiver participants, and Phase II will enroll up to 94 caregiver participants (please see “2.6 Data analysis and sample size estimation” section). Inclusion/exclusion criteria are as follows:

#### 2.2.1 Inclusion criteria

- Ability to understand and the willingness to sign a written informed consent document.
- Age 18 years and older.
- Serving as a primary caregiver for an adult patient (18 years and older) undergoing their 1^st^ allogeneic HSCT at the National Institutes of Health (NIH) Clinical Center during the four-week study period.
- Ability to read, speak, and understand English.
- Access to necessary resources for participating in online survey (i.e., computer, laptop, tablet, smartphone, internet access).

#### 2.2.2 Exclusion criteria

- Serving as a paid caregiver for the patient.
- Not agreeing to follow the study procedures.
- Recent use of immersive VR programs for stress relief and/or entertainment (more than 2 days/week within the past 3 months).
- Participation in another stress-reduction type interventional study within the past 3 months.
- Having a medical condition that is prone to frequent nausea or dizziness.
- Current or past history of seizure, chronic migraines, epilepsy, claustrophobia, panic disorder, post-traumatic stress disorder, generalized anxiety disorder, major depressive disorder or other known severe neurological or mental health disorders.
- Being sensitive to flashing light or motion.
- Having a balance disorder such as vertigo or cybersickness.
- Having another medical condition or injury that may prevent use of VR headset and/or VR software (e.g., visual or hearing problems, open sores, wounds, skin rash on face, or active infection).
- Self-reported diagnosis of Opioid, Cocaine, and/or Cannabis use disorder in the past year.

### 2.3 Interventional methods

#### 2.3.1 Nature-based immersive VR program (active VR; phase I/II)

Participants will receive a VR headset and be asked to perform the intervention for 20 minutes per days for four weeks. The intervention dose (20 minutes) was selected to decrease the risk of developing cybersickness. More prolonged exposure to VR is associated with a higher risk of cybersickness (30). In a systematic review of VR relaxation studies for the general population (28), 14 out of 19 studies administered a single-session format intervention for 20 minutes or less. There could be a novelty bias in VR research as it is an emerging technology in the health care field. Therefore, considering that caregiving stress is a type of chronic stress, we intend to set the intervention duration longer. The 4-week intervention duration was chosen based on two recent ongoing trials testing the effects of VR nature or relaxation for four weeks (31, 32).

For use in this study, we developed the nature-based immersive VR program software called “Nature VRelax.” All caregiver participants in Phase I and participants assigned to Active VR in Phase II will receive the Nature VRelax. The Nature VRelax contains 13 videos consisting of 360° high-definition video clips with nature sounds produced by Atmosphaeres ^1^. The video clips produced by Atmosphaeres have been tested in previous studies with different groups of patients and healthy adults; cybersickness, nausea, and dizziness were reported in several participants, but no serious adverse events occurred (33–36). Participants will select VR content from the pre-loaded 13 options on the VR headset, which fall into one of the following categories:

- Nature (11 videos): Nature scenes with pure nature sounds filmed in various locations (e.g., beach, river, meadow)
- Travel (2 videos): Scenes filmed in various historical sites and city attractions (e.g., London, Paris)

**Figure 2:**
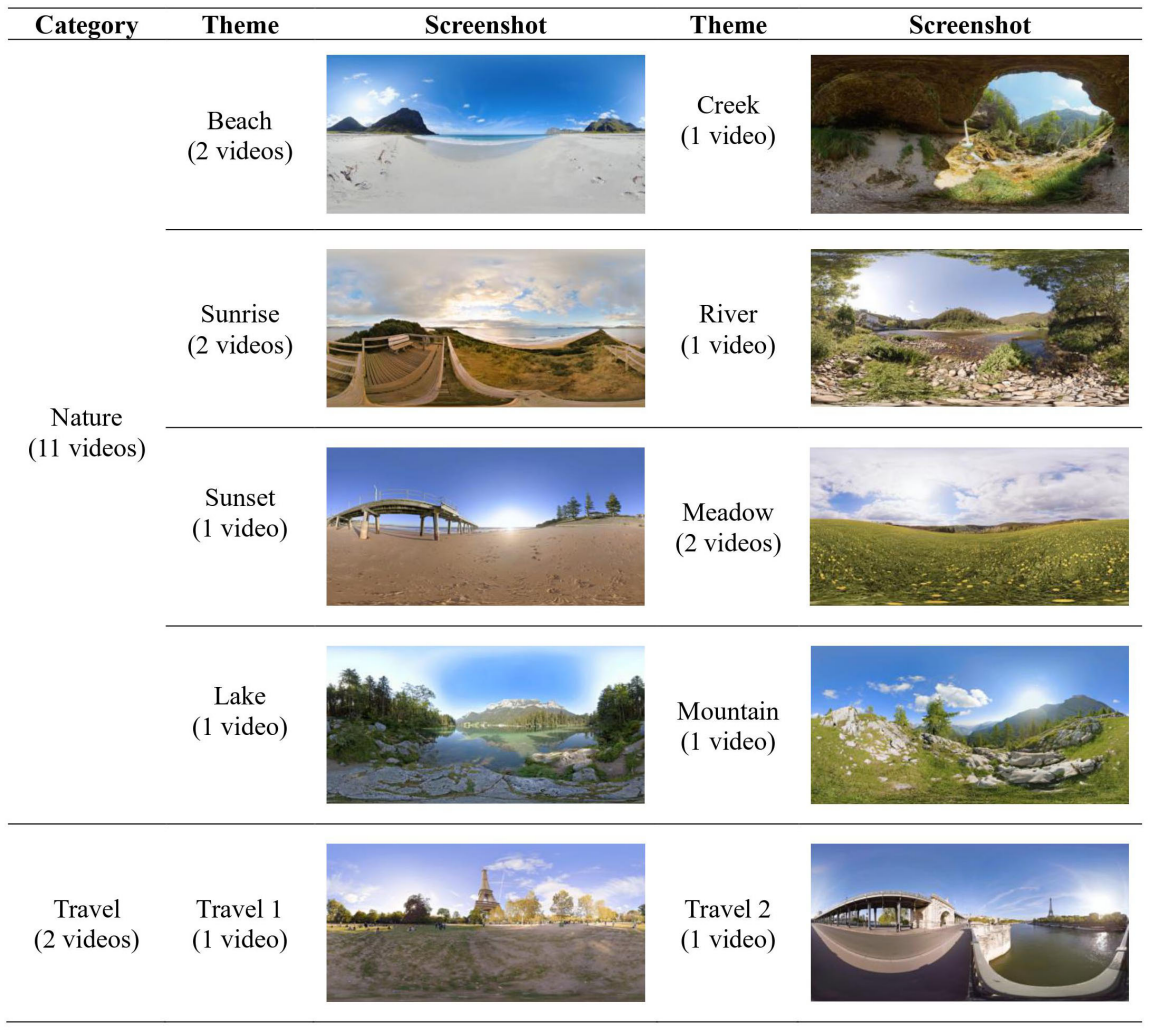
Themes and screenshots of virtual reality (VR) experiences available to study participants.

A complete list of VR experiences will be offered to study participants is listed in **Figure 2**.

#### 2.3.2 Nature-based non-immersive VR program (Sham VR; phase II only)

Participants assigned to Sham VR in Phase II will receive a nature-based non-immersive VR program. The Sham VR program contains 3D nature pictures with nature sounds pre-loaded on the VR headset. When the device is turned on, the nature photographs will be played automatically (one minute per photograph).

#### 2.3.3 VR headset

This study will use the Pico G3^2^ that delivers videos or computer-generated images and sounds to create an immersive environment. Pico G3 is a stand-alone head-mounted display that does not require a smartphone or a computer to run and is easy to use with an orientation-tracked controller or a handsfree control option. We will only provide the Nature VRelax pre-loaded videos on the VR headset and lock other external contents.

### 2.4 Procedures

A screening visit will be conducted for eligible caregivers who agree to participate. During the screening visit, eligibility will be assessed, and informed consent will be obtained if a caregiver meets all eligibility criteria. Time 0 baseline visit for all participants will occur within seven days of the HSCT infusion in a quiet room on the oncology/transplant unit at the NIH Clinical Center. During the Time 0 baseline visit, all participants will be trained on how to use the assigned VR program, including wearing the VR headset and navigating the VR videos. The research staff will guide the participants on the VR headset use to familiarize themselves with the device and allow them to test it on their own. Once the participants feel comfortable with the VR headset, they will experience the 15-minute video, which we developed for the initial testing purpose, under the supervision of the research staff. The research staff will monitor any issues or adverse events (AEs) associated with the VR program and will address any questions that the participants may have.

Following Time 0 baseline visit, participants will be instructed to use the Nature VRelax program for a maximum of 20 minutes, daily for four weeks. Once they complete practicing a 20-minute video, the VR program will be locked for the day and automatically reset at midnight. If they are interrupted during the session, they can resume or stop. Participants will be instructed to use the VR program in a static sitting position in a quiet place. Participants can connect a listening device (e.g., earphones, headphones, earbuds) to the VR headset as needed. The program can be used with or without a Wi-Fi connection. Participants will be informed that use usage of the program will be tracked on the VR headset. Participants will complete a daily use log. They will be instructed to discontinue use and notify the research staff immediately if they experience any unpleasant side effects, such as dizziness or nausea.

**Figure 3:**
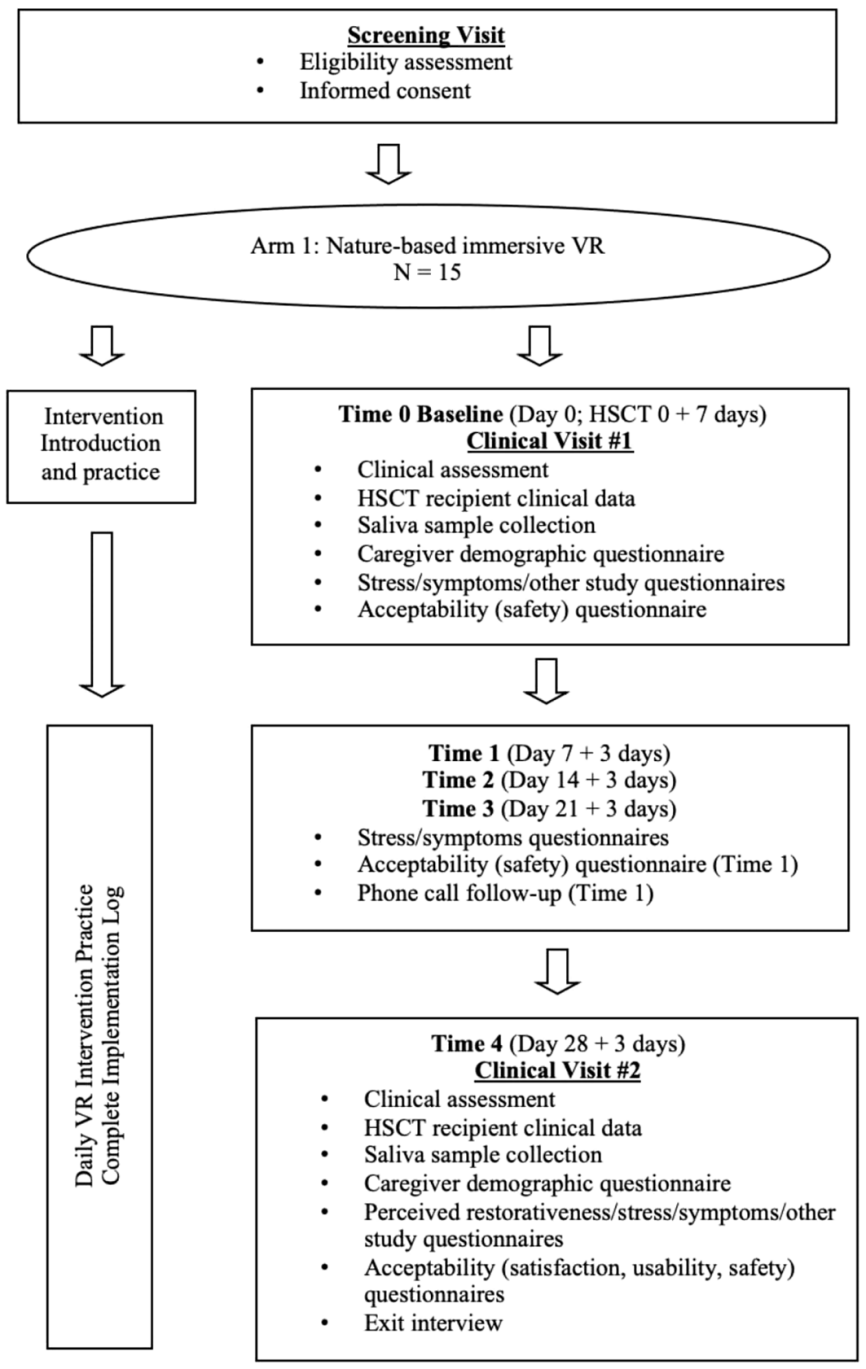
Research design and procedure (Phase I).

**Figure 4:**
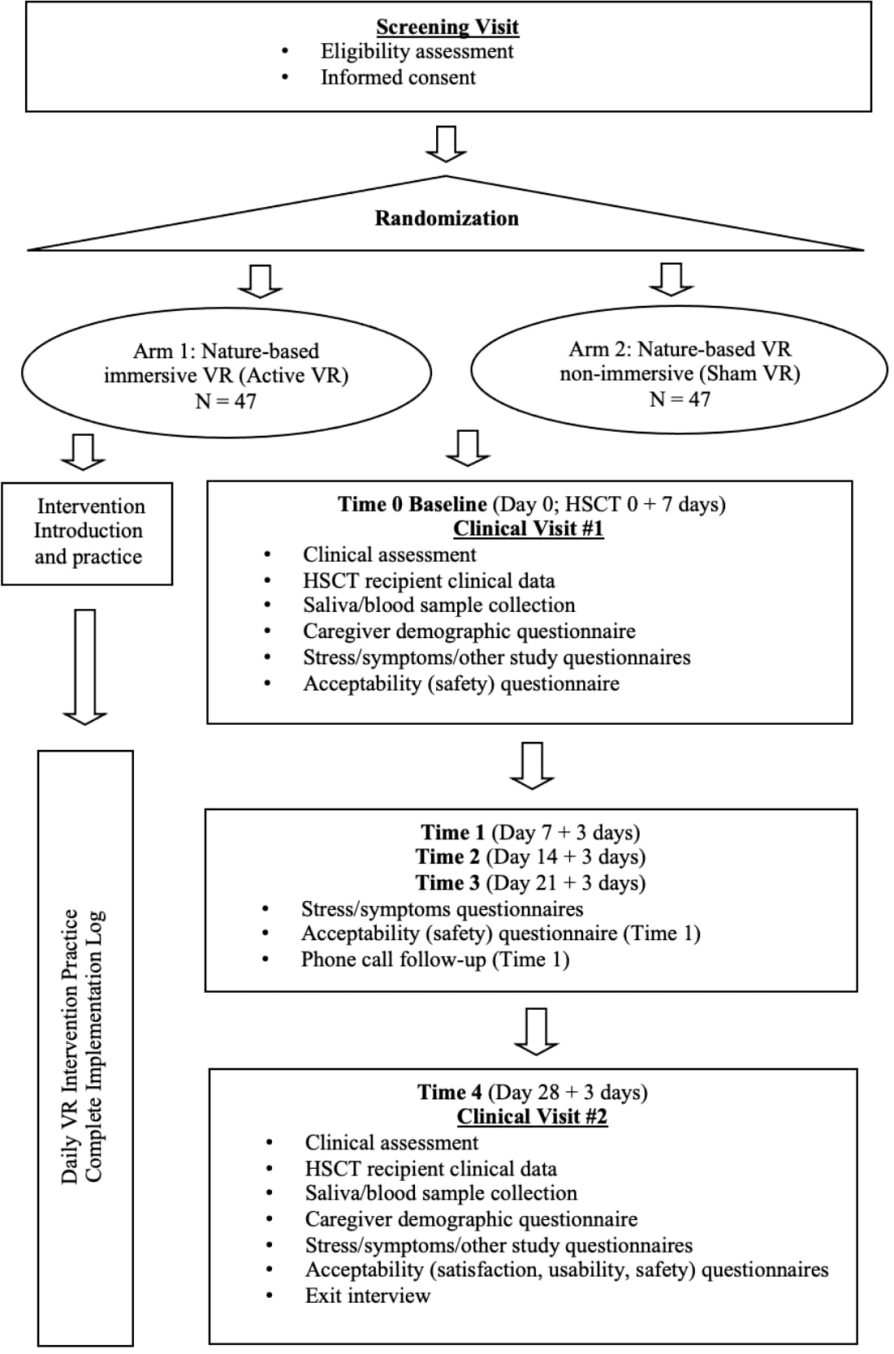
Research design and procedure (Phase II).

At each time point, all participants will be asked to complete a survey that will be delivered via the Research Electronic Data Capture (REDCap) system.^3^ The surveys at Time 0 (Day 0) and Time 4 (Day 28) will take about 20 to 30 minutes, and the surveys at Time 1 (Day 7), Time 2 (Day 14), and Time 3 (Day 21) will take about 3 to 5 minutes. The participants will be invited to a phone call follow-up at Time 1 and an exit interview at Time 4. Clinical assessments will be performed at Time 0 baseline visit and at Time 4 visit. Saliva samples will be collected three times at Time 0 baseline visit (pre-initial VR program, immediately post-initial VR program, 20 minutes post-initial VR program) and at Time 4 visit (pre-VR program, immediately post-VR program, 20 minutes post-VR program). To index cortisol awakening response, the participants will be instructed to collect three saliva samples (immediately post-awakening, 30 minutes post-awakening, nighttime) within three days of Time 0 and Time 4. In Phase II, blood samples will be collected at Time 0 baseline visit and at Time 4 visit. The schedule of activities or schema of Phase I and II are presented in **Figures 3**, **4**.

#### 2.4.1 Randomization

In Phase II, all caregiver participants will be randomized into either Active VR group or Sham VR group using permuted block randomization with an allocation ratio of 1:1. All investigators will remain blind to the randomization scheme until each study participant is deemed eligible and signs the consent form. Once each participant is randomly assigned, the participant and the research team members will be aware of the group assignment status.

### 2.5 Measures

#### 2.5.1 Feasibility and acceptability

Overall recruitment, retention, and adherence rates will be calculated as a part of the feasibility assessment. Satisfaction with the VR program will be measured using three items on a 5-point Likert scale (1 = extremely disagree, 5 = extremely agree) modified from the items used in Xu team’s VR exergame study (37). Usability of the VR program will be assessed using four items of the Usability scale from the Augmented Reality Immersion (ARI) Questionnaire rated on a 7-point Likert scale (1 = totally disagree, 7 = totally agree) (38). In each item, the phrase ‘AR application’ will be replaced with ‘VR program.’ Safety of the VR program will be assessed using the VR Sickness Questionnaire (VRSQ; 39), modified from the Simulator Sickness Questionnaire (40). The VRSQ consists of nine items on two components: oculomotor (four items); and disorientation (five items). Each item is rated on a 4-point scale (0 = none, 3 = severe). One open-ended question item is included asking if participants experienced any discomforts or side effects other than those specified in the VRSQ.

#### 2.5.2 Perceived restorativeness (phase I only)

Perceived restorativeness is measured using a modified version of the Perceived Restorativeness Scale (PRS) that reflects four key dimensions of the ART (41, 42). The measure consists of six items with three dimensions: being away, fascination, and coherence. Each item is rated on a 7-point Likert scale (1 = not at all, 7 = completely). Perceived restorativeness can be assessed by averaging the values of all six items. Tabrizian et al.’s immersive VR study using the same PRS measure reported Cronbach’s α of the measure as 0.76 (42).

#### 2.5.3 Perceived stress

Perceived stress will be measured using a 10-item modified version of the Perceived Stress Scale (PSS; 43). The measure evaluates the degree to which a participant has perceived life as unpredictable, uncontrollable, and overloading over the past week. Each item is rated on a 5-point Likert scale (0 = never, 4 = very often). Total PSS score is obtained by summing all items, where higher scores indicate higher levels of perceived stress. Gillani et al.’s study utilizing the same PSS measure for perceived stress over the past week for diabetic adults reported Cronbach’s α of 0.81 (44).

#### 2.5.4 PROMIS^®^ measures

The Patient Reported Outcome Measurement System (PROMIS^®^) is a system of highly reliable and precise measures of patient-reported health on physical, mental, and social well-being (45). Total sum scores are converted to T-scores, which are standardized scores with a mean of 50 and a standard deviation (SD) of 10. Higher scores represent more of the concept that is being measured. These measures will be delivered using Computer Adaptive Testing (CAT), if available. If CAT is not available, participants will complete the questionnaires using the short/fixed forms (4-10 items, each). The following PROMIS^®^ measures will be employed in this research:

- Fatigue: PROMIS^®^ Item Bank v1.0 - Fatigue
- Sleep disturbance: PROMIS^®^ Item Bank v1.0 - Sleep Disturbance
- Depression: PROMIS^®^ Item Bank v1.0 - Emotional Distress - Depression
- Anxiety: PROMIS^®^ Item Bank v1.0 - Emotional Distress - Anxiety
- Cognition: PROMIS^®^ Item Bank v2.0 - Cognitive Function
- Global health: PROMIS^®^ Scale v1.2 - Global Health

#### 2.5.5 NIH toolbox measures

The NIH Toolbox measures are a series of well-validated and reliable measures of patient self-reported health outcomes that assess cognitive, emotional, sensory, and motor functions (46). In this research, the NIH Toolbox Item Bank v3.0 - Loneliness (Ages 18+) - Fixed Form and the NIH Toolbox Item Bank/Fixed Form v2.0 - Self-Efficacy (Ages 18+) will be used to assess loneliness and self-efficacy. The loneliness measure is rated on a 5-point Likert scale ranging from 1 to 5, and the self-efficacy measure is rated on a 4-point Likert scale ranging from 1 to 4, with higher scores indicative of higher levels of the concept being measured. The self-efficacy measure will be delivered using CAT, and the loneliness measure will be assessed using the 5-item fixed form.

#### 2.5.6 Caregiver burden

Caregiver Burden will be measured using the Caregiver Reaction Assessment (CRA; 47). The CRA is a 24-item self-administered scale that measures the positive and negative effects of caregiving in five domains: caregiver esteem (7 items), impact on finances (3 items), impact on health (4 items), impact on schedule (5 items), and lack of family support (5 items). The items are rated on a 5-point Likert scale (1 = strongly disagree, 5 = strongly agree), and mean scores are calculated for each domain. Higher mean scores indicate greater caregiver burden with the exception of caregiver esteem. Mean scores range from 1 to 5. The measure is a valid and reliable for use in caregivers of cancer patients (48).

#### 2.5.7 Health-promoting behaviors

Health-promoting behaviors will be measured using the Health-Promoting Lifestyle Profile II (HPLP-II; 49). The HPLP-II is a is a self-administered 52-item instrument that measures the frequency of self-reported health behaviors. It consists of six subscales: health responsibility, interpersonal relations, nutrition, physical activity, spiritual growth, and stress management. It is a 4-point Likert type scale (1 = never, 4 = routinely) with possible scores ranging from 52 to 208. Higher scores indicate the more frequent engagement in health behaviors. The HPLP-II and its subscales have been performed reliably well in other studies of caregivers (50).

#### 2.5.8 Mutuality

Mutuality will be measured using the Family Caregiver Inventory (FCI) Mutuality Scale (51). The FCI scale is a 15-item questionnaire that addresses the relationship dimensions of reciprocity, love, shared pleasurable activities, and shared values between the caregiver and the care recipient. Each item is rated on a 5-point Likert scale (0 = not at all, 4 = a great deal). The average of all items across the four overarching themes produces a total mutuality score. The scale has demonstrated high levels of reliability, with Cronbach’s α ranging from 0.91 to 0.95 (51, 52).

#### 2.5.9 Biomarkers from saliva samples

Saliva samples will be collected by the passive drool method using an open tube and straw or saliva collection aid in a tube. Participants will allow the pooled drool to fall into a straw or a saliva collection aid that leads into a tube and will be asked to fill the tube with approximately 2mL of saliva. The following stress biomarkers will be analyzed from saliva samples: Cortisol, α-amylase, Osteocalcin, Oxytocin.

#### 2.5.10 Biomarkers from blood samples (phase II only)

Approximately 4 tablespoons of venous blood will be collected for cardiovascular, metabolic and inflammatory biomarkers. The following assays will be performed: nuclear magnetic resonance lipid analysis, multiplex cytokine immunoassay.

### 2.6 Data analysis and sample size estimation

For Phase I, a sample size of 12 is considered since this number is recommended to justify the study feasibility and precision of the mean and variance (53). To account for a 20% attrition rate, Phase I study will enroll up to 15 caregivers. To estimate a sample size for Phase II study, power analysis was performed based on the primary outcome. We used a two-sided hypothesis that the stress level will differ between Active VR group and Sham VR group. In order to detect a difference between Active VR group and Sham VR group, we will need 39 caregiver participants in each group and a total sample size of 78 based on an effect size of 0.6 and correlation of 0.80 between two time points from our previous stress reduction study (54), with 80% power and type 1 error of 0.05. To account for a 20% attrition rate, this study will enroll up to 94 caregivers.

In Phase I, feasibility, acceptability, and perceived restorativeness will be evaluated using descriptive statistics appropriate to the levels of measurement. Friedman test or linear mixed models will be used to test changes in stress measured weekly from Time 0 to Time 4. In Phase II, linear mixed repeated measures analysis will be used to analyze the effects of Active VR on stress compared to Sham VR. Any demographic covariates will be included in the model if significantly associated with the outcomes and significantly different between the two groups. All qualitative data, including written comments on open-ended questions and transcriptions of audio from the phone call follow-up and exit-interview, will be subjected to coding and qualitative data analysis.

## 3 Discussion

This two-phase study will be the first application of nature experience intervention using VR in family caregivers of HSCT recipients. The phase I study is a single-arm pre-post trial to evaluate the feasibility and acceptability of the four-week nature-based immersive VR program, and the phase II study is a prospective randomized controlled trial to test the effectiveness of the intervention on caregivers’ stress and symptoms. Existing interventions developed to relieve stress and symptoms in family caregivers usually required a great deal of time and effort from participants who may find it difficult to be away from their care recipients (50). Incorporating VR technology into stress reduction activities will provide family caregivers with immersive experiences by allowing them to experience real pleasant locations or realities without leaving the care recipient’s side. Studies have demonstrated novel applications of VR in various clinical scenarios. In particular, experiencing nature in a virtual environment was effective in reducing stress and psychological symptoms (e.g., pain and anxiety) (10, 13, 14). The application of nature environments implemented through VR is ideal for family caregivers with mobility and time constraints, who may have limited access to the nature environment in their real life and less opportunity to engage with nature. To our knowledge, limited studies have developed and tested interventions using VR in family caregivers. In this study, we expect that HSCT caregivers can take a break from their surroundings while experiencing restorative nature through immersive VR.

Despite the innovativeness and strengths of this study, a few limitations should be noted. First, only family caregivers of people receiving allogeneic HSCT at the NIH Clinical Center, a unique clinical research hospital setting providing care to those enrolled in research protocols with no charge to the research participants, are recruited to the study; thus, generalizability may be limited with respect to caregivers of other disease groups or patients receiving traditional care in general hospitals or clinics. Furthermore, this study recruits only English-speaking caregivers, which ultimately may limit generalizability to caregivers of other racial or ethnic backgrounds. In addition, in this study, once each participant is randomly assigned, the participant will be aware of the group assignment status. Therefore, the expectancy effect is not avoidable, which might influence participant behavior and the outcomes. A double-blind RCT design should be considered to get a more concise estimate of the specific effects of the intervention in the future. Lastly, this study will only examine the short-term effects (four weeks) of the intervention on stress and symptoms. Therefore, any longer-term effects of the nature-based VR intervention could not be captured in the present study. A more prolonged follow-up could further inform the long-term intervention effects in future trials and large-scale efficacy testing.

The nature-based immersive VR program has great potential to relieve stress and symptoms in family caregivers by providing the sense of being in real exposure to nature or outdoor environments, ultimately offering a source of respite for caregivers. Furthermore, by using objective biomarkers and validated self-report measures together, we will be able to assess the effects of our intervention comprehensively and accurately. Findings of this study will shed light on the use of nature-based VR intervention in caregiver populations across the spectrum of medical and psychological conditions. If proven feasible and effective, the nature-based VR program could be implemented to provide a convenient, attractive, and easily applied intervention to alleviate stress and symptoms in family caregivers at any time and place, differentiated from existing stress reduction interventions. Our findings will provide a foundation for future studies in diverse caregiver groups in terms of patient diagnosis and social determinants of health (e.g., age, sex, ethnicity). In addition, healthcare providers may recommend integrating the VR technology into clinical practice for both patients and their caregivers. The finding will lay the groundwork for the future study, in which we will aim to consider both patients and their caregivers to reduce their stress and stress-related symptoms.

## 4 Ethics and dissemination

This research protocol was approved by the NIH Clinical Center Institutional Review Board. Informed consent will be obtained from all study participants.

## Author contributions

LL: Conceptualization, Methodology, Supervision, Writing – original draft, Writing – review & editing, Data curation, Funding acquisition, Investigation, Resources. ES: Conceptualization, Data curation, Investigation, Methodology, Resources, Writing – original draft, Writing – review & editing. NF: Conceptualization, Data curation, Investigation, Writing – review & editing. CG: Data curation, Investigation, Resources, Writing – review & editing. RT: Data curation, Investigation, Resources, Writing – review & editing. LY: Formal analysis, Methodology, Validation, Writing – review & editing. JK-G: Project administration, Resources, Writing – review & editing. CS: Writing – review & editing. E-SN: Writing – review & editing. LS: Data curation, Resources, Writing – review & editing. SR: Data curation, Resources, Writing – review & editing. GW: Conceptualization, Funding acquisition, Supervision, Writing – review & editing.

## Footnotes

1
https://www.atmosphaeres.com

2
https://www.picoxr.com/global/

3
https://www.project-redcap.org/
